# Hyperbaric Oxygen Therapy Can Induce Angiogenesis and Regeneration of Nerve Fibers in Traumatic Brain Injury Patients

**DOI:** 10.3389/fnhum.2017.00508

**Published:** 2017-10-19

**Authors:** Sigal Tal, Amir Hadanny, Efrat Sasson, Gil Suzin, Shai Efrati

**Affiliations:** ^1^Sackler School of Medicine, Tel-Aviv University, Tel-Aviv, Israel; ^2^Radiology Department, Assaf Harofeh Medical Center, Zerifin, Israel; ^3^Sagol Center for Hyperbaric Medicine and Research, Assaf Harofeh Medical Center, Zerifin, Israel; ^4^Faculty of Medicine, Bar-Ilan University, Ramat Gan, Israel; ^5^WiseImage, Hod Hasharon, Israel; ^6^Research and Development Unit, Assaf Harofeh Medical Center, Zerifin, Israel; ^7^Sagol School of Neuroscience, Tel-Aviv University, Tel-Aviv, Israel

**Keywords:** hyperbaric oxygen, DTI, tractography, angiogenesis, MRI, perfusion, TBI, post-concussion

## Abstract

**Background:** Recent clinical studies in stroke and traumatic brain injury (TBI) victims suffering chronic neurological injury present evidence that hyperbaric oxygen therapy (HBOT) can induce neuroplasticity.

**Objective:** To assess the neurotherapeutic effect of HBOT on prolonged post-concussion syndrome (PPCS) due to TBI, using brain microstructure imaging.

**Methods:** Fifteen patients afflicted with PPCS were treated with 60 daily HBOT sessions. Imaging evaluation was performed using Dynamic Susceptibility Contrast-Enhanced (DSC) and Diffusion Tensor Imaging (DTI) MR sequences. Cognitive evaluation was performed by an objective computerized battery (NeuroTrax).

**Results:** HBOT was initiated 6 months to 27 years (10.3 ± 3.2 years) from injury. After HBOT, DTI analysis showed significantly increased fractional anisotropy values and decreased mean diffusivity in both white and gray matter structures. In addition, the cerebral blood flow and volume were increased significantly. Clinically, HBOT induced significant improvement in the memory, executive functions, information processing speed and global cognitive scores.

**Conclusions:** The mechanisms by which HBOT induces brain neuroplasticity can be demonstrated by highly sensitive MRI techniques of DSC and DTI. HBOT can induce cerebral angiogenesis and improve both white and gray microstructures indicating regeneration of nerve fibers. The micro structural changes correlate with the neurocognitive improvements.

## Introduction

Traumatic brain injury (TBI) is a significant public health concern in military and civilian populations (Chiu and LaPorte, 1993). The estimated number of TBI cases occurring each year is 10 million globally and 1.7–3.8 million in the United States alone. 75–90% of those are defined as mild TBI (mTBI) (Selassie et al., 2013; Marin et al., 2014).

The post-concussion syndrome (PCS) is a complex of symptoms consisting of headaches, dizziness, imbalance, vertigo, fatigue, changes in sleep pattern, neuropsychiatric symptoms (e.g., behavioral and mood changes, confusion), and cognitive impairments (in memory, attention, concentration and executive functions) (McCauley et al., 2005). PCS is most often described in the setting of mTBI, but may also occur after moderate and severe TBI. In Eight to Ninty percent of mTBI cases, the symptoms fade away in 7–10 days (McCrory et al., 2005; Hadanny and Efrati, 2016). Still, in 10–20 percent, PCS may persist for weeks or months due to structural and/or metabolic brain damage. Twenty-five to thirty-three percent of those retain a permanent brain injury and experience persistent PCS; the symptoms turn chronic and endure more than 6 months (Kashluba et al., 2004; Bazarian et al., 2005; Iverson, 2005; Sterr et al., 2006; King and Kirwilliam, 2011).

The sensitivity of classic anatomical brain imaging techniques, such as Computed Tomography (CT) and Magnetic Resonance Imaging (MRI), is generally not sufficient for detection of the pathophysiologic effects of mTBI. New techniques are increasingly utilized for objective evaluation of brain damage. Diffuse Tensor Imaging (DTI) can reveal the combination of axonal injury and secondary gliosis with local microvascular injury (Niogi and Mukherjee, 2010). Dynamic susceptibility contrast MR perfusion can demonstrate reduced cerebral blood flow (CBF), global and regional, as well as cerebral blood volume (CBV) (Tal et al., 2015).

The existing pharmacologic and non-pharmacologic treatments have mostly failed to elicit efficacious results in both the clinical symptoms and the pathophysiologic cascade leading to permanent brain injury (Hadanny and Efrati, 2016). In recent years, both basic (animal models) (Neubauer and James, 1998; Zhang et al., 2005; Vlodavsky et al., 2006; Chen et al., 2010; Huang and Obenaus, 2011; Lin et al., 2012; Efrati and Ben-Jacob, 2014) and clinical studies (Shi et al., 2003, 2006; Barrett et al., 2004; Golden et al., 2006; Wright et al., 2009; Harch et al., 2012; Boussi-Gross et al., 2013; Tal et al., 2015) have shown that hyperbaric oxygen therapy (HBOT) can improve PCS by targeting the basic pathological processes responsible for post-concussion symptoms (Hadanny and Efrati, 2016). In our previous study, it was evident that HBOT can induce brain angiogenesis, demonstrated by perfusion MRI with significant increase in CBF and CBV following HBOT along with significant cognitive improvement in patients post TBI (Tal et al., 2015).

The current study was aimed at evaluating the effects of HBOT on brain microstructure in chronic neurological deficiencies stemming from TBI. This has not been studied in humans so far.

## Methods

A retrospective analysis of patients suffering from chronic neurocognitive impairment due to TBI, treated at Sagol Center for Hyperbaric Medicine and Research at Assaf Harofeh Medical Center, Israel, between September 2013 and December 2015. The study was approved by the Institutional Review Board of the hospital (0030-15-ASF) and registered in the US National Institute of Health Clinical Trials registry (NCT02452619).

Inclusion criteria: Patients with chronic neurocognitive impairment started only after TBI, persisting over 6 months who underwent two MRI brain imaging (including DTI and DSC sequences) and two neurocognitive tests, pre- and post- hyperbaric oxygen therapy (HBOT). All patients applied for HBOT of their own volition. Patients with other neurological conditions were excluded from the study's cohort.

The classification of TBI was based on the American Congress of Rehabilitation Medicine (ACRM) and the Centers of Disease Control (CDC), where mTBI is defined as altered brain function engendered by external forces with one or more of the following: loss of consciousness with duration of 0–30 min, post-traumatic amnesia with duration of less than 24 h, and Glasgow Coma Scale grade of 13–15 (Malec et al., 2007). GCS score of 3–8, or post-traumatic amnesia of more than 7 days, or loss of consciousness for more than 24 h is classified as severe TBI; GCS score of 9–12, or post-traumatic amnesia of 1–7 days, or loss of consciousness between 30 min and 24 h, is classified as moderate TBI (Malec et al., 2007).

### Hyperbaric oxygen treatment

Patients were treated in a multiplace hyperbaric chamber (HAUX-Life-Support GmbH) for 60 daily hyperbaric sessions, 5 days a week. Each session consisted of 90 min of exposure to 100% oxygen at 2 ATA. Acceptable compression and decompression rates of 0.8 meter per minute were employed. Oxygen was delivered by tight masks.

### MRI scan protocol

All patients had MRI scans 1–2 weeks before and after HBOT. Imaging was done with a 3 Tesla system (20 channels, MAGNETOM Skyra, Siemens Medical Solutions) with a multichannel head coil as a receiver coil. The MRI protocol included the following sequences: T2 weighted, T1 weighted, FLAIR, susceptibility weighted imaging (SWI), dynamic susceptibility contrast enhancement (DSC), and diffusion tensor imaging (DTI). The MRI, DTI, and DSC sequences' parameters are detailed in the supplementary material (SI-1). The injected gadolinium (0.5 mmol/ml) dosage was 0.2 ml/kg/patient.

### MRI analysis

MRI analysis was performed by WiseImage (Hod Hasharon, Israel, http://www.wise-image.com).

### DSC analysis

Images were corrected for motion using SPM software (version 12, UCL, London, UK). DSC analysis was performed as described in previous studies (Østergaard et al., 1996a,b part I and II) using in-house software written in Matlab R2011 (Mathworks, Natick, MA). Detailed description is found in the Supplementary Material SI-1. In short, MR signal intensity was converted to Gd concentration, fitted to the gamma variate function and deconvolved on a voxel-by-voxel basis to calculate the CBF, CBV, and MTT (Mean Transient Time) maps. Smoothing of 8 mm full width at half maxima (FWHM) was performed on the perfusion maps using the SPM software.

### DTI analysis

Motion and Echo planar imaging (EPI) correction and regularization of the DWI volumes as well as calculation of DTI maps (MD = mean diffusivity, FA = fractional anisotropy, AD = axial diffusivity, RD = radial diffusivity maps) were done using ExploreDTI software (Leemans et al., 2009). Two analysis types were performed: voxel-based analysis and fiber tracking. Detailed description is found in the Supplementary Material SI-1. In short, paired *t*-test was performed using voxel-based analysis, generating statistical parametric maps. Fiber tracking was applied using Explore DTI software in order to plot 8 fiber tracts for each patient: Uncinate fasciculus (UF), Cingulum, inferior longitudinal fasciculus (ILF), and Inferior fronto-occipital fasciculus (IFOF), in both hemispheres. After the tracking procedure, a mask was created from the tracts matrices of all subjects in order to create a tract mask. Overall, 8 masks were created for each subject: four fiber tracts (UF, fornix, cingulum, ILF), in both hemispheres. The tract masks of the different patients were registered to a tract mask of one patient.

### Cognitive assessment

The assessment of cognitive functions was done by NeuroTrax computerized cognitive tests (NeuroTrax Corp., TX) (Dwolatzky et al., 2003). The neurocognitive battery by Neurotrax is a validated computerized cognitive evaluation, which was specifically designed to TBI patients. These tests evaluate various aspects of brain function and incorporate Verbal Memory (immediate and delayed recognition), Non-Verbal Memory (immediate and delayed recognition), Go-No-Go Response Inhibition, Problem Solving, Stroop Interference, Finger Tapping, Catch Game, Staged Information Processing Speed (single digit, two-digit and three-digit arithmetic), Verbal Function, and Visual Spatial Processing. Cognitive index scores were computed from the normalized outcome parameters for the following domains: executive function, memory, attention, speed of information processing, visual spatial, verbal function and motor skills (Thaler et al., 2012; Achiron et al., 2013; Zur et al., 2015). The verbal domain score was excluded because only 8 patients (53%) had reliable calculated verbal domains. A global cognitive score was computed as the average of all index scores for each individual.

The NeuroTrax data were uploaded to the NeuroTrax central server, and outcome parameters were automatically calculated using a software blind to diagnosis or testing site. To account for the effects of age and education on cognitive performance, each outcome parameter was normalized and fit to an IQ-like scale (mean = 100, S.D. = 5) according to age and education. The normative data used by NeuroTrax consist of test data from cognitively healthy individuals in controlled research studies at more than 10 sites (Doniger, 2014).

Specifically, the patients received two different versions of the NeuroTrax test battery before and after HBOT so as to produce minimal learning effects upon repeated administration. Test-retest reliability for those versions was found to be high (Schweiger et al., 2003; Melton, 2005). Each cognitive domain score was calculated out of 3–5 different tests. It had 3 different forms of each test - out of which we used one for pre exam and another for the post test. This is a strong feature of these tests as it reduces the “learning effect” with a good test/retest validity. The fact that each index is referred to more than one test-score ensures that the index is associated with a cognitive domain score rather than with a test-dependent score.

### Statistical analysis

In addition to the MRI analysis described above, continuous data were expressed as means ± standard errors. The normal distribution for all variables was tested by means of the Kolmogorov-Smirnov test. The mean differences between cognitive index scores before and after HBOT were analyzed using two-tailed paired *t*-tests or a Wilcoxon signed-rank test. The alpha level was set to 0.05. Data were statistically analyzed using SPSS software (version 22.0).

## Results

### Patients

Fifteen patients with chronic cognitive impairment due to TBI who were treated at the Sagol Center for Hyperbaric Medicine and Research between September 2013 and December 2015 fulfilled the inclusion criteria.

The mean age was 35.8 ± 3.5 years (21–70), and 53% (8/15) were males. All patients had documented traumatic brain injury 6 months to 27 years (mean 6.7 ± 2.1 years) prior to HBOT. Seven patients (46.7%) suffered from moderate to severe TBI, and 8 (53.3%) suffered from PCS after mTBI. See patients' baseline characteristics in Table 1. Baseline standard MRI findings for each of the patients are summarized in SI-2.

**Table 1:** Patients' baseline characteristic.

**Age (years)**		35.8 ± 3.5
**Sex**		
	Males	8 (53.3%)
	Females	7 (46.7%)
**Time from trauma (years)**		6.7 ± 2.1
**Severity of trauma**		
	Mild	8 (53.3%)
	Moderate	2 (13.3%)
	Severe	5 (33.4%)
**Trauma type**		
	MVA	13 (86.7%)
	Fall	1 (6.7%)
	Assault	1 (6.7%)
**Medications**		
	SSRI	3 (20%)
	Benzodiazepines	1 (6.7%)
	Opiates	2 (13.3%)

### Neurocognitive function

HBOT induced a considerable improvement in the global cognitive score, with a mean change of 8.1 ± 1.5 and a relative change of 9.6 ± 1.9% (*p* = 0.0001). Memory, executive functions and information processing speed showed the most striking improvements (>15% relative change) with mean changes of 10.5 ± 2.4 (*p* = 0.001), 11.3 ± 2.7 (*p* = 0.0001) and 13.1 ± 2.7 (*p* = 0.0001), respectively. Attention increased by 16.1 ± 6.3% post HBOT but did not reach statistical significance (*p* = 0.06). There were no differences in neurocognitive scores (mean and relative changes) of patients taking SSRI/Opiates/Benzodiazpeines drugs compared to patients without them (*p* > 0.2).

**Figure 1:**
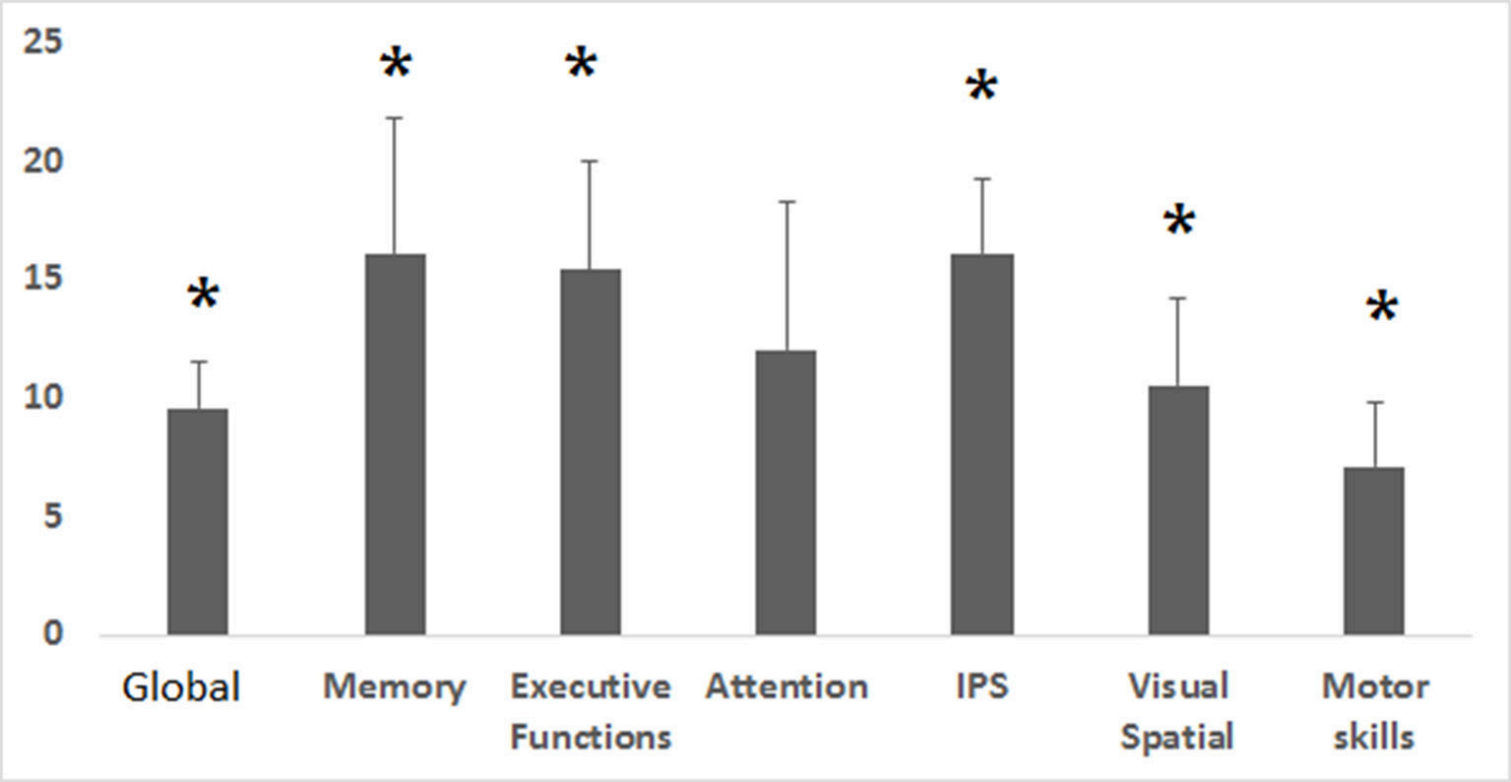
Cognitive indices relative changes post HBOT. Relative change in the corresponding cognitive indices after HBOT. Relative change was calculated by (post HBOT-pre HBOT)/Pre HBOT. IPS, Information processing speed. ^*^p < 0.05.

The effect of HBOT on the patients' cognitive functions is summarized in Figure 1 and Table 2.

**Table 2:** Cognitive indices at baseline, and after Hyperbaric Oxygen Therapy (HBOT).

	**Baseline**	**Post HBOT**	**Mean change**	**Sig**.	**Sig. with time as covariate**
Global	88.2 ± 2.5	96.4 ± 2.5	8.2 ± 1.5	^*^0.0001	**0.0004**
Memory	82.2 ± 5.3	92.7 ± 4.7	10.5 ± 2.4	^*^0.001	**0.008**
Executive Functions	83.9 ± 3.8	95.2 ± 3.4	11.3 ± 2.7	^*^0.001	**0.002**
Attention	88.1 ± 3.5	96.3 ± 2.9	8.2 ± 4.0	0.062	0.105
IPS	84.3 ± 3.3	97.4 ± 3.8	13.1 ± 2.7	^*^0.0001	**0.001**
VSP	96.6 ± 4.0	105.3 ± 3.1	8.7 ± 3.0	^*^0.01	**0.04**
Motor skills	92.3 ± 4.1	98.2 ± 3.8	5.8 ± 2.0	^*^0.0009 (W)	

*Data are expressed as means ± standard errors. IPS, Information processing speed; VSP, Visual spatial processing; W, Wilcoxon signed-rank test. Bold values indicated Statistically significant p < 0.05*.

### Brain microstructure integrity changes

**Figure 2:**
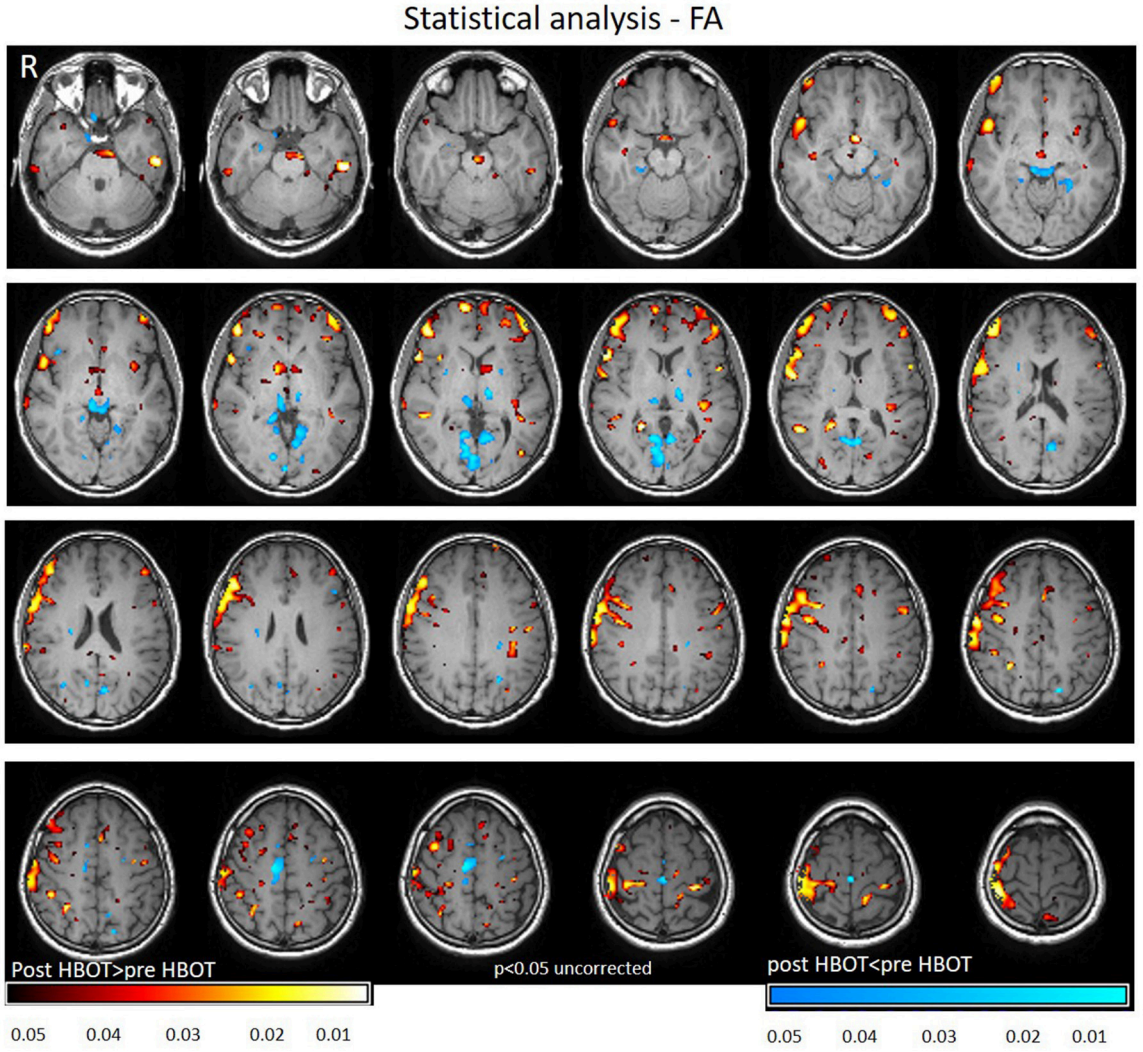
Average DTI normalized delta change in FA maps. Yellow and red areas show a statistically significant increase in FA (*p* < 0.05).

**Figure 3:**
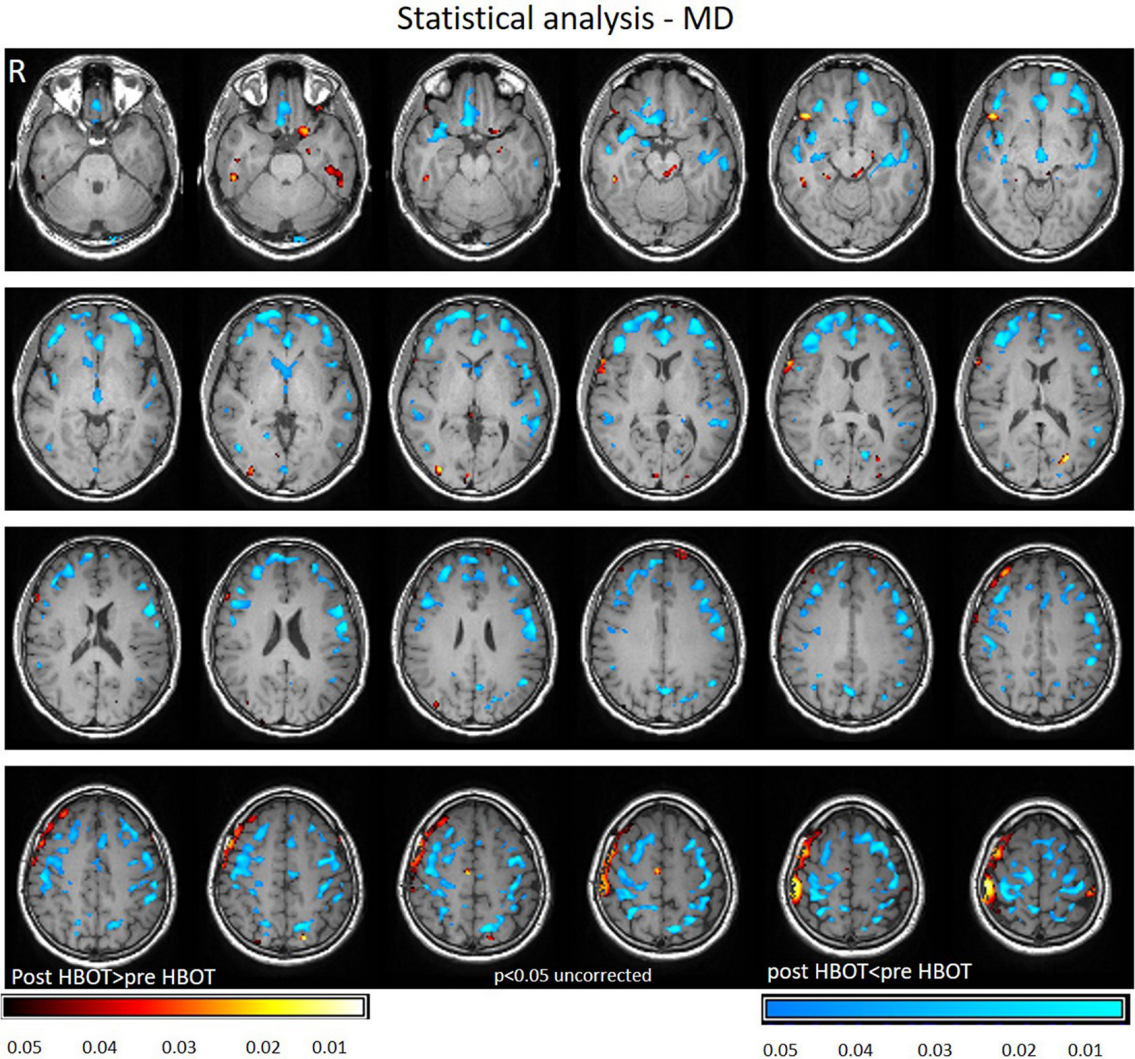
Average DTI normalized delta change in MD maps. Blue areas mark statistically significant decrease in MD (*p* < 0.05).

Voxel-based DTI analysis was compared before and after HBOT using paired *t*-test. FA and MD whole brain maps are depicted in Figures 2, 3, and show the statistically significant increase in FA (yellow in Figure 2) and decrease in MD (blue in Figure 3) average values.

### Regional changes in brain microstructure integrity

Statistically significant increase in FA was found in regions related to motor function (internal capsule, midbrain), association fiber tracts inferior fronto-occipial fasciculus (IFOF), inferior longitudinal fasciculus (ILF), superior longitudinal fasciculus (SLF), Cingulum and in the genu of the Corpus Callosum.

**Figure 4:**
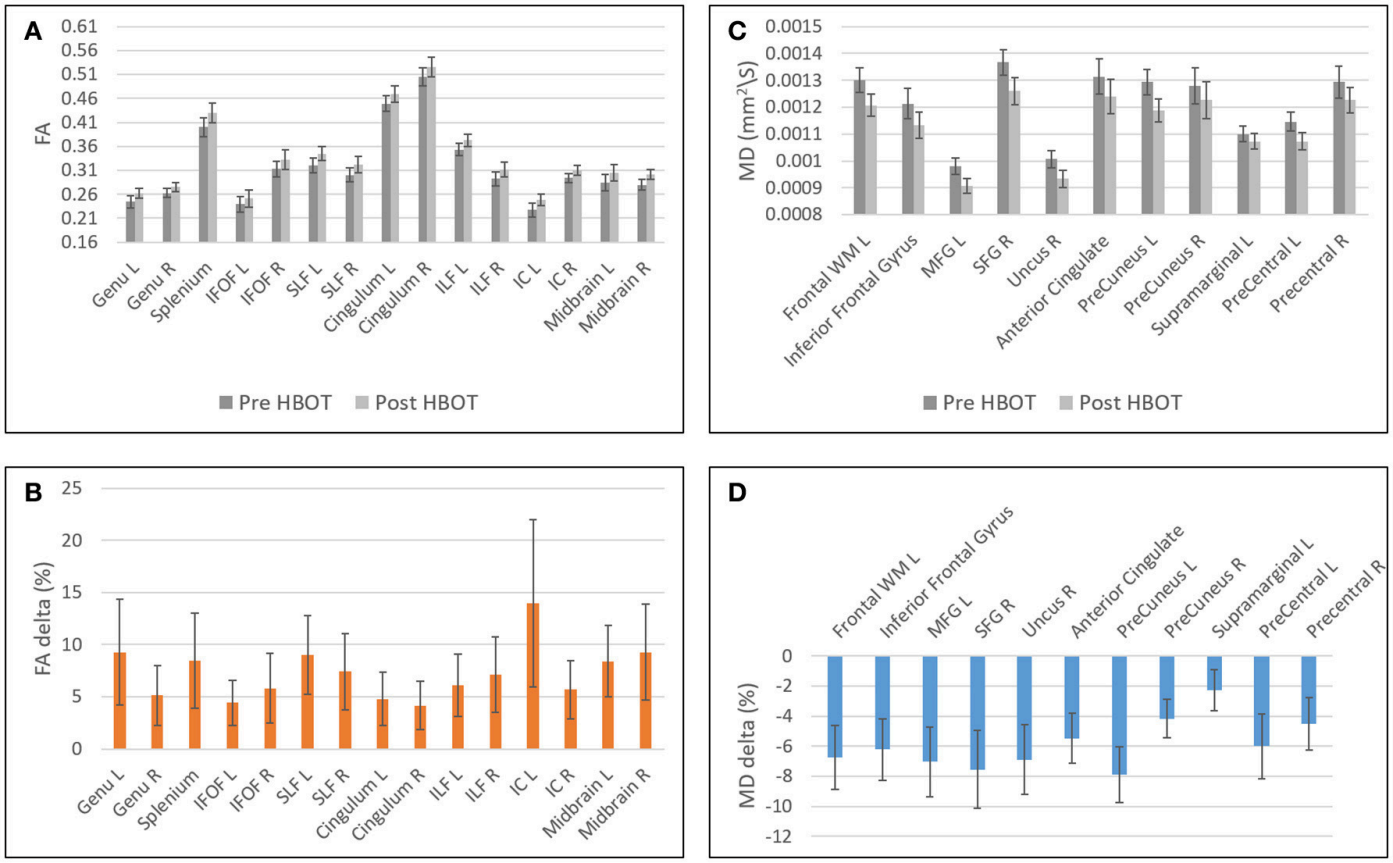
Graphs of FA and MD averages and standard error in statistically significant clusters. **(A)** Averages of FA before and after HBOT. **(B)** Normalized delta of FA maps. **(C)** Averages of MD before and after HBOT. **(D)** Normalized delta of MD maps.

Decrease in FA was found in areas related to the visual system (superior colliculi, calcarine sulcus) and other cognitive areas (thalamus, and posterior cingulate gyrus). Graphs of FA in significant clusters are presented in Figures 4A,B.

Decrease in MD was found in the frontal lobe (anterior cingulate gyrus, posterior orbital gyrus, Precuneus, superior frontal gyrus, Uncinate fasciculus, and frontal lobe white matter, left middle frontal gyrus, precentral gyrus). Graphs of MD in significant clusters are presented in Figures 4C,D.

### White matter tracts integrity

**Figure 5:**
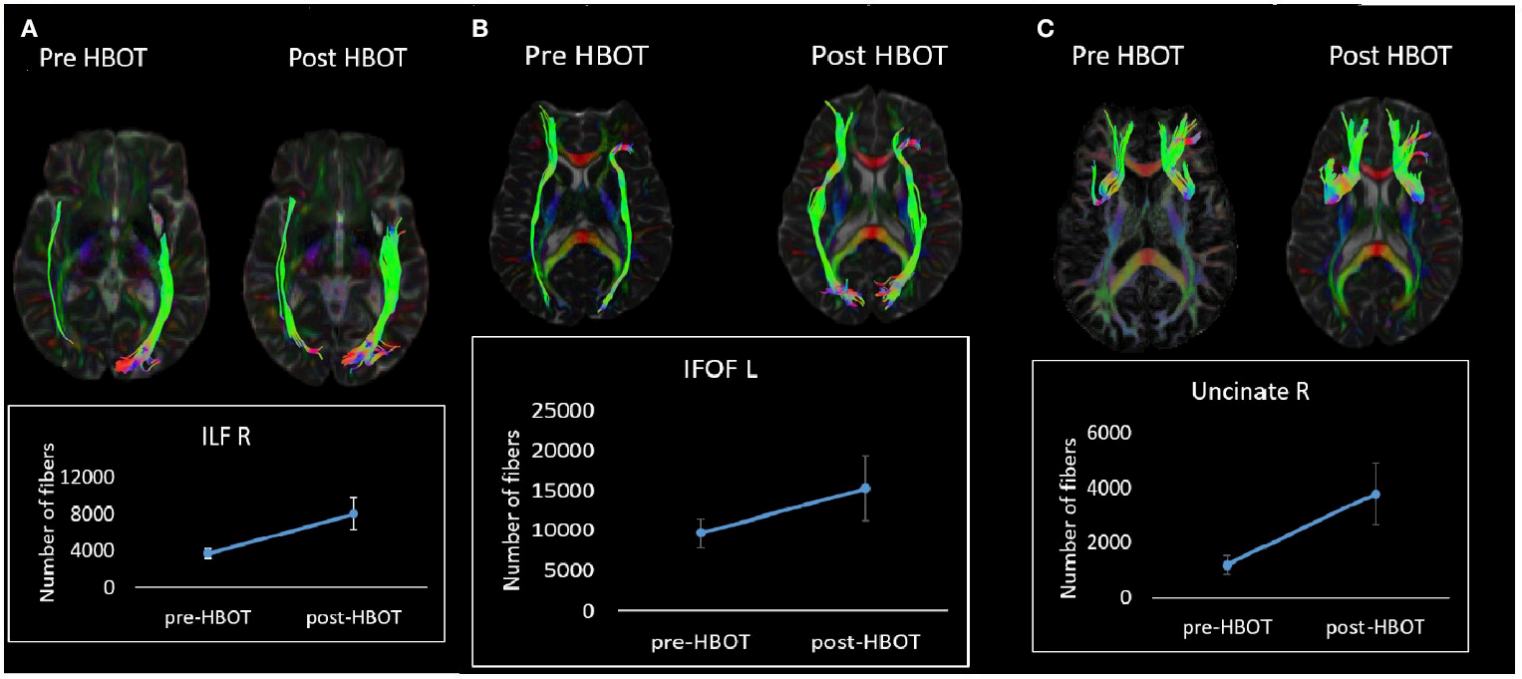
White matter tractography change in a single patient. **(A)** Fibers number increase in the right ILF tract. **(B)** Fibers number increase in the left IFOF tract. **(C)** Fibers increase in the right Uncinate tract.

Fiber tracking analysis revealed a statistically significant increase in number of fibers in the left cingulum (*p* = 0.03) (Figure 5) and in the right ILF following HBOT (*p* = 0.029) and in the right Uncinate fasciculus (*p* = 0.04) (Figure 5).

### Increased brain perfusion

Voxel-based DSC analysis was compared before and after HBOT using paired *t*-test. Average CBV and CBF and delta whole brain maps are depicted in Figure 5, and show the increase in both CBF and CBV post HBOT.

### Regional changes in brain perfusion

Statically significant increases in CBF involved frontal white matter (including corpus callosum), association fibers (SLF, IFOF), motor function-related structures (corona radiata, midbrain, and cerebellum) and structures related to memory function (temporal GM and fornix).

**Figure 6:**
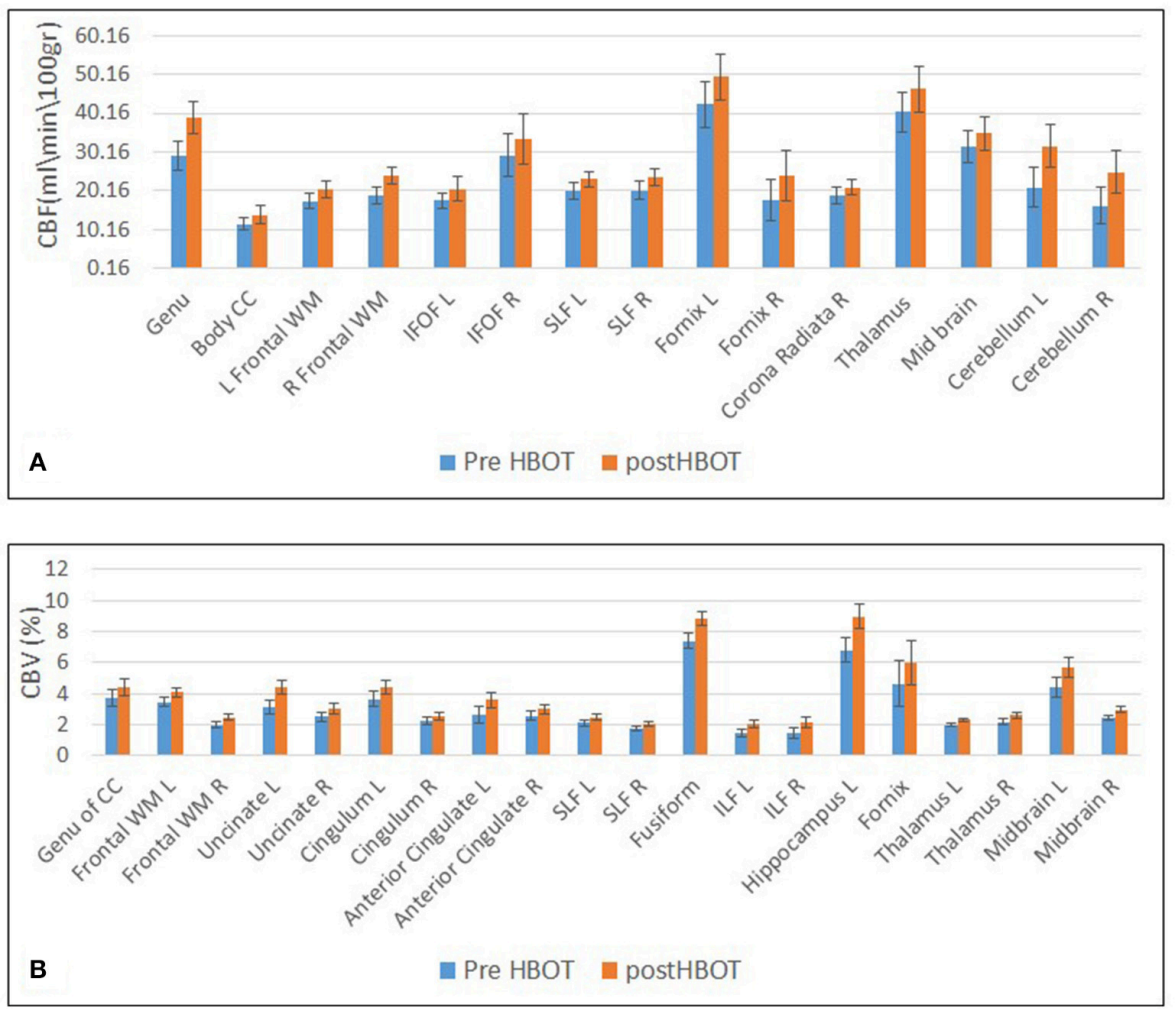
Graphs of CBF and CBV averages and standard error in statistically significant clusters. **(A)** Averages of CBF before and after HBOT. **(B)** Averages of CBV before and after HBOT.

**Figure 7:**
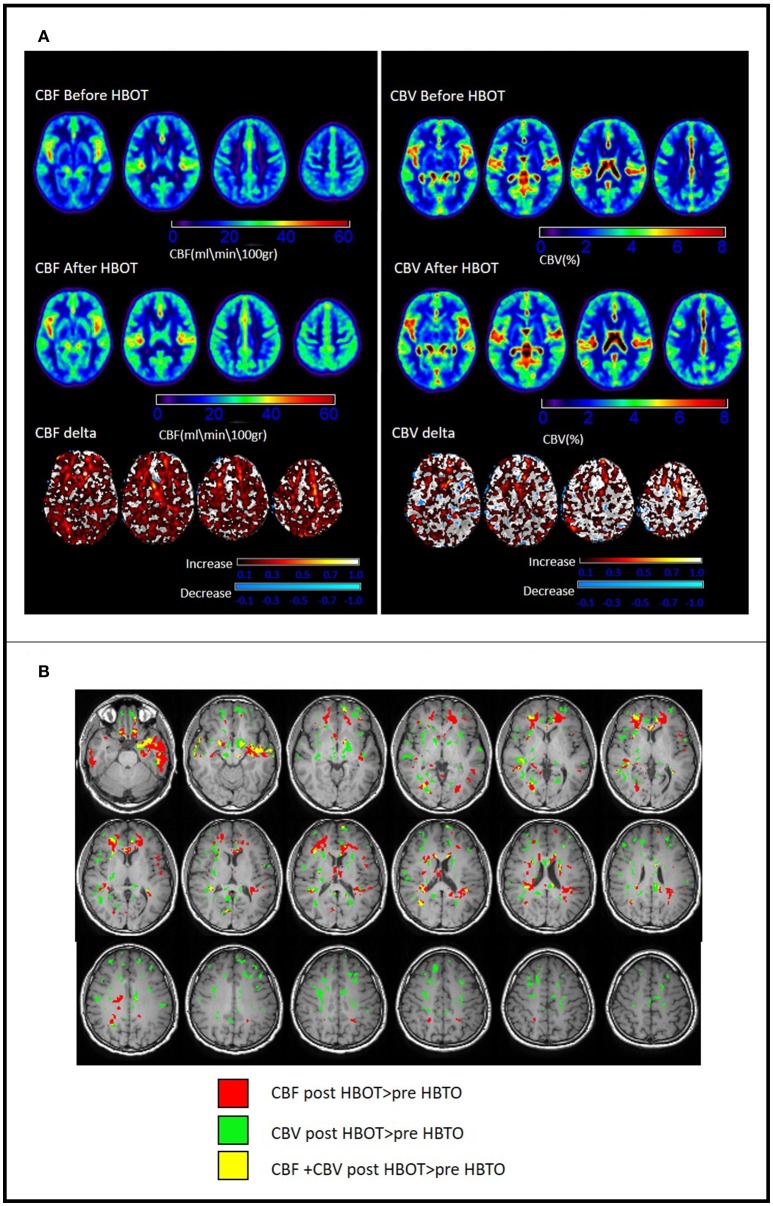
Changes in brain perfusion (CBF and CBV) post HBOT. **(A)** Average DSC maps pre and post HBOT and DSC normalized delta maps. Top row: CBF and CBV pre-HBOT. Middle row: CBF and CBV maps post-HBOT. Bottom row: normalized delta maps, showing diffuse increases in CBF and CBV post-HBOT. **(B)** Significant CBF and CBV normalized delta changes post HBOT. Areas of maximal statistically significant increase in perfusion.

Statistically significant increase in CBV was found in frontal white matter (including Uncinate fasiculus and Corpus Callosum), frontal gray matter (anterior cingulate), regions related to sensory-motor function and executive functions (including the thalamus and midbrain) association fiber tracts (SLF, ILF and cingulum) and regions related to memory function (hippocampus and fornix) (Figures 6, 7).

Most of the anatomic structures that presented a significant increase in CBF also exhibited an increase in CBV (Figure 6).

## Discussion

The current study shows, for the first time in humans, that HBOT can induce brain microstructure recovery in TBI patients. Brain recovery encompassed gray and white matter areas, white matter tracts and angiogenesis. Post HBOT, FA increased and MD decreased in the DTI sequence, CBV and CBF increased in the DSC (perfusion) sequence, along with improved cognitive functions. Recovery was induced in the late chronic stage of TBI (6.7 ± 2.1 years post injury).

Previous studies using DTI have reported that patients suffering from TBI may still have microstructural damage months to years after the initial injury (Kraus et al., 2007; Yuan et al., 2007; Lipton et al., 2008; Sugiyama et al., 2009; Hartikainen et al., 2010; Niogi and Mukherjee, 2010; Murugavel et al., 2014; Perez et al., 2014; Li et al., 2016). DTI characterizes the diffusion of water in the tissues, thus it indicates microstructural density, spacing, and orientational organization of cell membranes, including myelin (Assaf and Pasternak, 2008; Alexander et al., 2011). Animal studies of brain plasticity, revealed that decrease in MD and increase in FA correlates with synaptophysin (a marker of synaptic vesicles), glial fibrillary acidic protein (GFAP; a marker of astrocyte activation), and brain-derived neurotrophic factor (BDNF; a marker of neuronal growth that facilitates learning) (Sagi et al., 2012). With regards to white matter, it was found that myelin density estimated by DTI can accurately predict the actual myelin density seen/measured by electron microscopy (Sepehrband et al., 2015). The injury can be demonstrated by increased water diffusion as measured by MD and reduced directionality of diffusion as measured by FA, suggesting that either axonal injury or disruption of myelination could have altered brain connectivity (Kraus et al., 2007; Yuan et al., 2007; Lipton et al., 2008; Sugiyama et al., 2009; Hartikainen et al., 2010; Niogi and Mukherjee, 2010; Murugavel et al., 2014; Perez et al., 2014; Li et al., 2016). Moreover, FA changes appear to correlate with the severity of the clinical presentation (Benson et al., 2007; Yuan et al., 2007). The observed decrease in FA values may reflect the barriers to axoplasmic transport, the local accumulation of apoptosis in organelles, and secondary Wallerian degeneration in the white matter, while the increased MD values may be the result of vasogenic cerebral edema.

Significant injury foci were reported in dedicated pathways involved in the transmission of efferent and afferent information, such as the corpus callosum, internal capsule, SLF, ILF, SFO, superior frontal gyrus, insula, and fornix (Yuan et al., 2007; Caeyenberghs et al., 2010). Importantly, the microstructure injuries depicted by the DTI imaging markers, FA and MD, correlated with objective measures of general and cognitive functioning (Benson et al., 2007; Assaf and Pasternak, 2008; Sugiyama et al., 2009; Caeyenberghs et al., 2010; Hartikainen et al., 2010; Alexander et al., 2011; Sagi et al., 2012; Wada et al., 2012; Arenth et al., 2014; Haberg et al., 2015; Sepehrband et al., 2015; Li et al., 2016). Disorders in reaction time, executive functions, information processing speed, attention and memory were correlated with axonal lesions in different areas. Moreover, in two recent longitudinal studies, FA values increased in patients with favorable outcome within 6–12 months, while no DTI changes registered in patients with unfavorable outcome (Sidaros et al., 2008; Hartikainen et al., 2010).

In the current study, for the first time in humans, DTI changes of chronic TBI were evaluated before and after HBOT. The increase in FA and decrease in MD post HBOT, together with cognitive function improvement of patients in the late chronic stage of TBI, suggest that brain microstructure recovery can be induced by HBOT.

DTI values, FA and MD, were found in our study to correlate with the improvements in cognitive functions in concordance with previous studies (Sugiyama et al., 2009; Wada et al., 2012; Arenth et al., 2014; Haberg et al., 2015). Memory, executive function and information processing speed were all significantly improved. In correlation with these cognitive improvements, MD decreased in most of the frontal lobe white matter, such as the prefrontal cortex that enables executive control (Miller and Cohen, 2001) and the anterior cingulate gyrus involved in error detection, especially in a Stroop task (Bush et al., 2000). Also, FA increased in most of the long association fibers critical for proper cognitive function:

- SLF–Bi-directional connection of the hemispheric frontal, parietal, temporal and occipital lobes. The SLF plays an important role in high brain functions, particularly language, reflected in information processing speed and executive function tasks (Heilman et al., 1970; Rocha et al., 2005; Sasson et al., 2013). In correlation with those changes, there was a significant improvement in neurocognitive test results in both information processing speed (IPS) and executive functions (EF) (i.e., IPS: 13.1 ± 2.7, *p* < 0.0001; EF: 11.3 ± 2.7, *p* < 0.001).
- ILF–Connection between the temporal and occipital lobes on the same hemisphere. The ILF is known to play an important role in visual memory (Bauer and Trobe, 1984; Shinoura et al., 2007). In correlation with those changes, there was a significant improvement in the memory index, which includes a visual memory task (i.e., Memory: 10.5 ± 2.4, *p* < 0.001).
- Cingulum – A cluster of white matter fibers projecting from the cingulate gyrus in the frontal lobe to the entorhinal cortex in the temporal lobe. The cingulum has been tightly associated with memory disorders (Charlton et al., 2006; Sepulcre et al., 2009). The memory correlates also with the changes in the cingulum.
- Genu of the Corpus Callosum-The largest white matter structure in the brain. It connects the left and right cerebral hemispheres and facilitates interhemispheric communication. Integrity of the corpus callosum is linked to information processing speed and episodic memory (Bucur et al., 2008; Lockhart and DeCarli, 2014). The improvement in information processing speed and memory indices may also correlate with the improvement in the genu of the corpus callosum.

Mechanisms of neuroplasticity and cellular repair by HBOT have been suggested in many animal studies (Hadanny and Efrati, 2016). These include enhanced mitochondrial function and cellular metabolism, improved blood brain barrier and inflammatory reactions, reduced apoptosis, alleviation of oxidative stress, increased levels of neurotrophins and nitric oxide, and up-regulation of axonal guidance agents (Efrati et al., 2013; Efrati and Ben-Jacob, 2014). Moreover, the effects of HBOT on neurons may be mediated indirectly by glial cells. HBOT may also promote neurogenesis of endogenous neural stem cells (Efrati et al., 2013; Efrati and Ben-Jacob, 2014). HBOT may enable the metabolic change simply by supplying the missing oxygen/energy needed for these regeneration processes (Efrati et al., 2013; Efrati and Ben-Jacob, 2014). The ability of HBOT to induce angiogenesis was demonstrated in several different pre-clinical studies (Mu et al., 2011; Lin et al., 2012; Lee et al., 2013; Hu et al., 2014). Hu et al. have demonstrated that HBOT-induced neurogenesis is mediated by ROS/HIF-1α/β-catenin pathway (Hu et al., 2014). In the current study, it is demonstrated that HBOT can induce neuroplasticity in humans even years after the acute insult.

Along with the structural changes, HBOT induces angiogenesis, as shown by the increase of CBF and CBV in this study as well as in our previous study (Tal et al., 2015). The injured areas in the brains post TBI experience hypoxia and hypoperfusion, which serve as a rate-limiting factor for any regenerative process (Graham and Adams, 1971; Graham et al., 1978; Stein et al., 2004; Kim et al., 2010; Ostergaard et al., 2014). HBOT-induced angiogenesis has been amply confirmed in pre-clinical models and can be deduced from brain SPECTs of patients post stroke and post TBI even years after the acute insult (Lin et al., 2012; Boussi-Gross et al., 2013; Efrati et al., 2013; Peng et al., 2014; Duan et al., 2015). The generation of new microvessels renders the local environment non-hypoxic, thus able to induce brain plasticity, enhance neurogenesis and synaptogenesis and foster functional recovery (Chen et al., 2003; Jiang et al., 2005). Unsurprisingly, CBV and CBF increased in the long association fiber tracts discussed above, including corpus callosum, association fibers (SLF, IFOF) and cingulum. Angiogenesis and increased perfusion to the malfunctioning tissue, seen in DSC, serve as infrastructure for the regenerative process and the preservation of newly generated metabolic functioning of the axonal microstructure seen in DTI.

Our study has several limitations. The major one is related to lack of control group. However, one can hardly expect any significant changes in DSC and DTI values or neurocognitive improvement to occur spontaneously years after the acute insult. The cognitive improvement seen here is in line with our earlier randomized controlled trial on patients suffering from mild TBI. In our previous randomized control study it was clearly demonstrated that the control group had no neurocognitive improvement (same cognitive tests used in the current study) or significant change of in brain perfusion measured by SPECT 1-5 years after the acute insult (Boussi-Gross et al., 2013). Nevertheless, one can hardly expect any significant changes in DSC and DTI values or neurocognitive improvement occurring spontaneously years after the acute insult. In addition, a previous randomized controlled trial with a control group showed neurocognitive effects and brain perfusion improvement using SPECT (Boussi-Gross et al., 2013).

## Conclusion

HBOT can induce cerebral angiogenesis and recovery of brain microstructure in patients with chronic cognitive impairments due to TBI months to years after the acute injury. The increased integrity of brain fibers correlates with the functional cognitive improvement. The mechanism by which HBOT can induce brain neuroplasticity can be demonstrated by highly sensitive perfusion MRI and DTI. Further studies, using DTI - MRI, are needed in order to gain better understanding of the neuroplasticity effect of HBOT in a larger cohort of patients with different types of brain injuries.

## Ethics statement

This study was carried out in accordance with the recommendations of Assaf Harfoeh Medical Center Institutional Review Board (0030-15-ASF) with written informed consent from all subjects. All subjects gave written informed consent in accordance with the Declaration of Helsinki. The protocol was approved by the Assaf Harfoeh Medical Center Institutional Review Board (0030-15-ASF).

## Author contributions

Conceived and designed the experiments: AH, ST, and SE. Performed the experiments: AH, ST, GS, and SE. Analyzed the data: AH, ES, and SE. Contributed analysis tools: AH, ST, ES, and SE. Wrote the paper: AH, ST, GS, ES, and SE.

### Conflict of interest statement

The authors declare that the research was conducted in the absence of any commercial or financial relationships that could be construed as a potential conflict of interest.

## Abbreviations

CBF: cerebral blood flow
CBV: cerebral blood volume.
