# Supplementary material for: Hyperbaric Oxygen Therapy Can Induce Angiogenesis and Regeneration of Nerve Fibers in Traumatic Brain Injury Patients
Source: Front Hum Neurosci. 2017 Oct 19;11:508. doi: 10.3389/fnhum.2017.00508 (PMC5654341; doi:10.3389/fnhum.2017.00508)
Supplement: Supplementary file 1 [file DataSheet1.PDF]

SI-1:

MRI sequences parameters:

- **DTI:** 30 diffusion weighted images were scanned with different gradient directions (b=1000) and one volume without diffusion weighting, with the following parameters: TR=10,300 ms, TE=89 ms, Voxel size = 1.8X1.8 mm, Matrix = 128 X 128, No. of slices = 63, Slice thickness = 2.2mm.
- ***DSC:*** 50 T2*-weighted gradient-echo echo planar imaging (EPI) volumes were acquired, 2 repetitions before a bolus injection of Gadolinium-DTPA (Gd-DTPA), 48 repetitions after injection of Gd-DTPA. Sequence parameters: TR=2,300 ms, TE= 40ms, flip angle = 30°, Voxel size = 1.8 x1.8 mm, Matrix = 128x128, No. of slices = 25, Slice thickness = 3.9 mm.

(The injected gadolinium (0.5 mmol/ml) dosage was 0.2 ml/kg/patient)

MRI Analysis

DSC analysis steps:

1. Conversion of signal intensity to concentration of Gd-DTPA with respect to time:

$$C_m(t) = -K * \ln(\frac{S(t)}{S_0})$$

where Cm(t) is the measured concentration of Gd-DTPA with respect to time, K is a proportionality constant that is inversely proportional to the TE and depends on the MR scanner, S(t) is the MRI signal intensity with respect to time, and So is the baseline MRI signal before the presence of Gd-DTPA and after a steady-state magnetization has been achieved [1].

2. Arterial Input function measurement: the AIF was measured in a region of interest (ROI) outlined manually, in voxels with increased signal on the Cm(t)

images in the 13th \ 14th volume. Voxels with maximal Cm(t) lower than 2 and higher than 4 were excluded. The ROI was outlined in the same anatomic regions for both scans. A similar AIF plot was defined for both scans - before and after HBOT (excluding voxels with undesired plot shape).

3. Gamma fitting of AIF and Cm: The AIF and Cm(t) were fitted to the gamma variate function using the following equation [1].

$$AIF_{fit}(t)\ or\ C_{fit}(t) = -K(x-\Delta)^{\alpha} * e^{-\frac{x-\Delta}{B}} * F_{step}(x-\Delta)$$

where AIFfit(t) and Cfit(t) are the fitted AIF(t) and Cm(t) curves, respectively, K is a constant, x is the image number, Δ is the delay between image 0 and the arrival of the bolus (a positive number), α and B are gamma variate parameters, and Fstep is a step function defined by:

$$F_{step} = \begin{cases} 1 & for\ (x-\Delta) \geq 0 \\ 0 & for\ (x-\Delta) < 0 \end{cases}$$

4. Singular value decomposition (SVD) deconvolution: The fitted AIF was used to calculate C(t) (the tissue response to an instantaneous arterial bolus) using SVD deconvolution, as done by Ostergaard et al. (1996). In short, the values for the AIF and Cm(t) curves can be written in vector notation as C = AIF−1 • Cm, where C represents the matrix of the deconvolved C(t) curve. This equation can be solved using the SVD technique, whereby the matrix AIF is decomposed into three matrices AIF = U • W • VT. The inverse of AIF can be calculated as AIF^{-1} = V • [diag(1/wj)] • UT, where [diag(1/wj)] represents the reciprocals of the diagonal elements of W. When calculating AIF^{-1}, problems arise when W contains singular values (i.e., wj = 0 or is close to 0), which

causes the curve C(t) to oscillate. Therefore, we used a cutoff threshold of 10% [2]

5. Calculation of CBV was performed based on the fitted Cm(t) and AIF:

$$CBV = \frac{k}{\rho} * \frac{\int C_m(t)\,dt}{\int AIF(t)\,dt}$$

where k = (1 − HCTLV)/(1 − HCTSV) corrects for the fact that the hematocrit in large vessels (HCTLV was set to 0.45) is larger than the hematocrit in small vessels (HCTSV was set to 0.25) (1) and ρ is the density of brain tissue (1.04 g/ml) [1].

6. Calculation of CBF was performed using the following equation:

$$\frac{CBV}{CBF} = \frac{\int C(t)\,dt}{C_{max}}$$

where C(t) is the concentration of Gd-DTPA in a tissue region and Cmax is the maximum of this curve [1].

7. MTT (Mean Transient Time) was calculated [3]

$$MTT = \frac{CBV}{CBF}$$

Voxel-based analysis

Spatial normalization and statistical analysis were performed using the SPM software (version 12, UCL, London, UK).

For DTI, spatial normalization was performed for each patient based on the mean DWI image with similar contrast to the template used in SPM (ICBM template, based on T1 contrast). The normalization parameters were applied on the DTI maps. Spatial smoothing with kernel size of 8mm full width at half maximum (FWHM) was applied.

For DSC, spatial normalization was performed for each patient based on the first T2* image. The normalization parameters were applied on the DSC maps. Spatial smoothing with kernel size of 8mm full width at half maximum (FWHM) was applied.

Paired t-test was performed using voxel-based analysis, generating statistical parametric maps. The statistical parametric maps (p values) are presented superimposed on a T1 image from a single subject to permit informative anatomical reference. Using the statistical parametric maps for each of the statistical paired t-tests, we report significant voxels ($p<0.05$).

Fiber tracking

Fiber tracking was applied using ExploreDTI software. The principal eigenvectors and FA were used to generate the fiber coordinates, terminating at voxels with FA lower than 0.2 or following tract orientation change higher than 30°. Fibers that passed through a manually chosen seed region of interest (ROI) were plotted. The fibers were plotted as streamlines. The masks obtained were overlaid over the B0 image. Overall 8 fibers tracts were plotted for each subject: four temporal projections (Cingulum, uncinate fasciculus (UF), inferior longitudinal fasciculus (ILF) and inferior fronto-occipital fasciculus (IFOF)), in both hemispheres.

1. The Cingulum: Three ROIs were used in order to reconstruct the cingulum bundle. One seed ROI was drawn in the axial plane in the body of cingulum, second ROI in mid sagittal plane in the posterior part of the cingulum. A third was drawn in a more lateral sagittal slice in the temporal part of the cingulum adjacent to the hippocampus. Where necessary, in order to eliminate fibers which are not part of the cingulum, a “no-fiber” plane was drawn in a coronal plane posterior to the cingulum and in an axial plane dorsal to the cingulum.
2. The UF: two ROIs were used to reconstruct the UF in a coronal plane. First ROI was drawn in the temporal part of the UF, and a second ROI was drawn in the frontal part of the UF. Where necessary, in order to eliminate fibers which are not part of the UF, a “no-fiber” plane was drawn in an axial slice dorsal to the frontal part of the UF.
3. The ILF: two ROIs were used to reconstruct the ILF in a coronal plane. First ROI was drawn in the occipital (posterior) part of the ILF. Second ROI was drawn in the temporal (anterior) part of the ILF. Where necessary, in order to eliminate fibers which are not part of the ILF, a “no-fiber” plane was drawn in a coronal plane anterior to the ILF.
4. The IFOF: two ROIs were used to reconstruct the IFOF in a coronal plane. First ROI was drawn in the occipital (posterior) part of the IFOF. Second ROI was drawn in the frontal (anterior) part of the IFOF. Where necessary, in order to eliminate fibers which are not part of the IFOF, a “no-fiber” plane was drawn in a coronal plane anterior to the IFOF.

References

1. Smith AM, Grandin CB, Duprez T, Mataigne F, Cosnard G (2000) Whole brain quantitative CBF, CBV, and MTT measurements using MRI bolus tracking: implementation and application to data acquired from hyperacute stroke patients. J Magn Reson Imaging 12: 400-410.
2. Ostergaard L, Sorensen AG, Kwong KK, Weisskoff RM, Gyldensted C, et al. (1996) High resolution measurement of cerebral blood flow using intravascular tracer bolus passages. Part II: Experimental comparison and preliminary results. Magn Reson Med 36: 726-736.
3. Ostergaard L, Weisskoff RM, Chesler DA, Gyldensted C, Rosen BR (1996) High resolution measurement of cerebral blood flow using intravascular tracer bolus passages. Part I: Mathematical approach and statistical analysis. Magn Reson Med 36: 715-725.
