# Supplementary material for: Hyperbaric Oxygen Therapy Can Induce Angiogenesis and Regeneration of Nerve Fibers in Traumatic Brain Injury Patients
Source: Front Hum Neurosci. 2017 Oct 19;11:508. doi: 10.3389/fnhum.2017.00508 (PMC5654341; doi:10.3389/fnhum.2017.00508)
Supplement: Supplementary file 2 [file DataSheet2.PDF]

SI-2: MRI findings

Patient#	Fluid Attenuation Inversion Recovery (FLAIR)	Susceptibility Weight Imaging (SWI)	T1+ Gadolinium
1	Periventricular white matter lesions	Occipital hemosiderin deposit	No enhancement
2	Normal	Normal	No enhancement
3	Normal	Normal	No enhancement
4	Normal	Normal	No enhancement
5	Left frontal lobe chronic post traumatic brain lesions	Multiple corpus callosum hemosiderin deposits and left frontal lobe hemosiderin deposits	No enhancement
6	Normal	Normal	No enhancement
7	right temporal chronic post traumatic lesions	Multiple cerebellar hemosiderin deposits<br>right temporal hemosiderin deposits	No enhancement
8	Left frontal lobe and temporal lobe chronic post traumatic brain lesions and left chronic subdural hematoma	Left frontal and temporal lobes hemosiderin deposits	No enhancement
9	Normal	Normal	No enhancement
10	Normal	Normal	No enhancement
11	Normal	Normal	No enhancement
12	Bilateral frontal and temporal chronic post traumatic lesions	Bilateral frontal and temporal multiple hemosiderin deposits	No enhancement
13	Periventricular white matter lesions	Normal	No enhancement
14	Left temporal and insular chronic post traumatic lesions	Left temporal and insular hemosiderin deposits	No enhancement
15	Bilateral temporal and left insular chronic post traumatic lesions	Bilateral temporal and left insular multiple hemosiderin deposits	No enhancement
